# Suppression of Plant Resistance Gene-Based Immunity by a Fungal Effector

**DOI:** 10.1371/journal.ppat.1000061

**Published:** 2008-05-09

**Authors:** Petra M. Houterman, Ben J. C. Cornelissen, Martijn Rep

**Affiliations:** Plant Pathology, Swammerdam Institute for Life Sciences, University of Amsterdam, Amsterdam, The Netherlands; Johns Hopkins University School of Medicine, United States of America

## Abstract

The innate immune system of plants consists of two layers. The first layer, called basal resistance, governs recognition of conserved microbial molecules and fends off most attempted invasions. The second layer is based on Resistance (*R*) genes that mediate recognition of effectors, proteins secreted by pathogens to suppress or evade basal resistance. Here, we show that a plant-pathogenic fungus secretes an effector that can both trigger and suppress *R* gene-based immunity. This effector, Avr1, is secreted by the xylem-invading fungus *Fusarium oxysporum* f.sp. *lycopersici* (Fol) and triggers disease resistance when the host plant, tomato, carries a matching *R* gene (*I* or *I-1*). At the same time, Avr1 suppresses the protective effect of two other *R* genes, *I-2* and *I-3*. Based on these observations, we tentatively reconstruct the evolutionary arms race that has taken place between tomato *R* genes and effectors of Fol. This molecular analysis has revealed a hitherto unpredicted strategy for durable disease control based on resistance gene combinations.

## Author Summary

In agriculture, the most environmentally friendly way to combat plant diseases is to make use of the innate immune system of plants, for instance by crossing into crop varieties polymorphic resistance genes that occur in natural populations of the crop plant or its close relatives. Plant pathogens, however, have co-evolved with their host plants and have developed ways to overcome the immune system. To effectively make use of components of the plant immune system, it is therefore important to understand the co-evolution of plants and their pathogens at the molecular level. For the interaction between a fungal pathogen and tomato, this paper presents a breakthrough in this respect. A small protein secreted by some strains of the fungus *Fusarium oxysporum* was found to suppress the activity of two disease resistance genes of tomato. However, a third resistance gene specifically targets this suppressor protein and renders the plant fully resistant against fungal strains that produce it. With this insight, together with knowledge of the genetic variation in the pathogen population, a combination of resistance genes is suggested that is expected to confer durable resistance in tomato against Fusarium wilt disease.

## Introduction

Long periods of co-evolution of plants and microorganisms have led to complex mechanisms of attack and defence, involving the innate immune system of plants and virulence factors of pathogens [1]. The first layer of plant defence, called basal immunity, is based on recognition of conserved microbial molecules but can be suppressed by microbial virulence factors known as “effectors”. Plants respond to this suppression by employing a second layer of defence, Resistance (*R*) gene-based immunity, which relies on recognition of effectors [2]. In turn, at least bacterial pathogens have found ways to manipulate or evade this second layer of defence [3]. It is unclear to what extent this capacity exists in eukaryotic plant pathogens like oomycetes and fungi.

Like bacteria, many plant-pathogenic fungi secrete proteins that are recognized by *R*-genes [4],[5]. One of these fungi is *Fusarium oxysporum*, a common soil inhabitant. It propagates asexually and is mostly harmless. However, pathogenic and host-specific clonal lines have evolved that cause severe diseases in crops, such as banana, cotton, cucumber, melon and tomato [6],[7]. Many of these diseases are caused by colonisation of the water-conducting xylem system of the roots followed by upward growth through xylem vessels, with wilting and death as a dramatic result. Strains of *F. oxysporum* that cause wilt of tomato plants are grouped in *forma specialis* (f.sp.) *lycopersici*. Several polymorphic resistance (*R*) genes have been identified in the tomato gene pool that each confer resistance against a subset of *F. oxysporum* f.sp. *lycopersici* (Fol) strains. These are *I* (for *Immunity*), *I-1*, *I-2* and *I-3* [8]. Races of Fol are named historically according to the *R* gene that is effective against them: the *I* gene and the (unlinked) *I-1* gene are effective against race 1, race 2 overcomes *I* and *I-1*, but is stopped by *I-2*, while race 3 overcomes *I*, *I-1* and *I-2* but is blocked by *I-3* [9]. Race 1 strains have been further divided into subgroups based on whether or not they are able to (partially) overcome *I-2* or *I-3* [9],[10].

Based on the gene-for-gene hypothesis [11], it is assumed that disease resistance conferred by *R* genes in tomato requires ‘matching’ avirulence (*AVR*) genes in Fol. The *I* gene originates from *Solanum [Lycopersicon] pimpinellifolium* and resides on chromosome 11 [12],[13], while the *I-1* gene is located on chromosome 7 in another wild relative of tomato, *Solanum [Lycopersicon] pennellii* [14]. The *I-2* gene has been cloned and encodes an R protein of the common NB-LRR class [15]. The *I-3* gene has not yet been cloned [16], but the matching *AVR* gene has: it encodes a small protein, Six1 (“Secreted in xylem 1”), which is secreted by Fol during colonization of the xylem system [17] and contributes to fungal virulence [9]. Six1 is now called Avr3 to indicate its gene-for-gene relationship with the *I-3* resistance gene.

We describe here the identification and analysis of a second avirulence factor of Fol, Avr1. Surprisingly, this protein does not only act as an avirulence factor in conjunction with the *I* gene, but also suppresses disease resistance mediated by *I-2* and *I-3*.

## Results/Discussion

### Identification of Avr1

**Figure 1:**
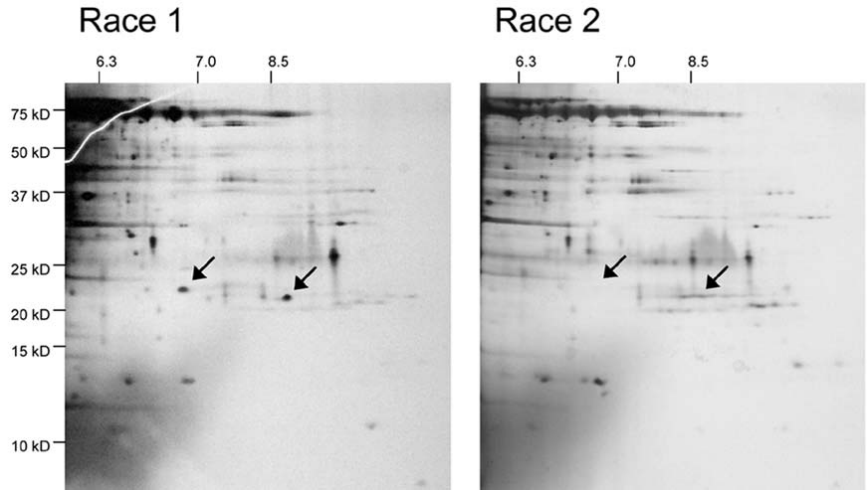
Fol race 2 does not secrete Avr1/Six4. Proteins present in xylem sap of susceptible tomato plants infected with race 1 strain Fol004 (left panel) or race 2 strain Fol002 (right panel) were isolated and separated with 2-dimensional gel electrophoresis. Positions of isoelectric point markers are indicated at the top; positions of molecular weight markers are indicated on the left. The arrows in the left panel point to the two spots previously shown to contain Avr1 (Six4) [18]; the arrows in the right panel point to the corresponding (empty) positions. The right spot in the left panel likely represents a more extensively N-terminally processed form of Avr1 [18].

**Figure 2:**
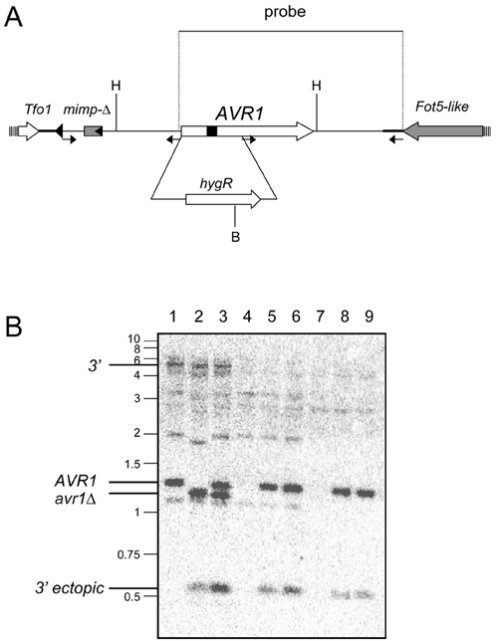
The *AVR1* locus, gene deletion and complementation. A) The *AVR1* open reading frame (ORF; open arrow) is interrupted by a single intron (black box) [18] (accession AM234064). The ORF is flanked 714 bp upstream by a copy of the transposon *Tfo1* (striped arrow represents the end of the transposase ORF; triangle represents the inverted repeat), 485 bp upstream by a partial miniature impala repetitive element (*mimp*-Δ, grey box; triangle represents inverted repeat) and downstream by a *Fot5*-like repetitive element (the transposase ORF ends 541 bp downstream of the *AVR1* ORF and is shown as a grey arrow). The small arrows denote the primers used to construct an *AVR1* disruption construct and an *AVR1* expression cassette for transformation to Fol (see Materials and methods). The insertion of a hygromycine resistance (*hygR*) cassette to create an *AVR1* knock-out mutant is shown (not drawn to scale). The position of the probe and the restriction sites used for Southern blot analysis are indicated; H: *Hin*dIII, B: *Bam*HI. B) Southern blot confirming *AVR1* disruption and ectopic insertion of *AVR1*. A Southern blot of genomic DNA digested with *Hin*dIII and *Bam*HI was probed with a 1.4 kb probe encompassing the *AVR1* ORF and 3′ sequences as indicated in Fig. 2A. The *AVR1* locus in race 1 strain Fol004 (lane 1) is visible as a 1.25 kb *Hin*dIII band containing the ORF (*AVR1*) and a band of ∼5 kb containing sequences 3′ of the ORF (3′). In the race 1 *avr1*Δ strain (lane 2), replacement of the ORF with the disruption cassette through homologous recombination led to the expected replacement of the 1.25 *Hin*dIII band with a 1.1 kb *Bam*HI-*Hin*dIII band containing part of the ORF and part of the disruption cassette (*avr*1Δ). Transformation of the *AVR1* expression cassette to the *avr1*Δ strain (lane 3) led to reappearance of the *AVR1* band. Race 2 strain Fol007 (lane 4) and race 3 strain Fol029 (lane 7) do not contain AVR1 (the *AVR1* and 3′ bands are absent). Transformation of the *AVR1* expression cassette to these strains (lanes 5 and 6: race 2 transformants; lanes 8 and 9: race 3 transformants) leads to appearance of the 1.25 kb *Hin*dIII *AVR1* band as well as a 0.56 kb *Hin*dIII-*Bam*HI band (3′ ectopic) that comprises sequences 3′ of the *AVR1* ORF until the *Bam*HI site at the 3′ end of the expression cassette (which is not present in the genomic locus but corresponds to the end of the probe shown in Fig. 2A). Note that in the *avr1*Δ strain (lane 2) the 0.56 kb band indicative of ectopic insertion is also present, indicating that this strain contains an additional copy of the disruption cassette. The additional, weaker bands are probably due to 104 bp of non-coding sequence of the Fot5-like transposon present at the 3′ end of the probe (thick line next to the grey arrow in Fig. 2A) – there are seven copies of this sequence in the latest release of the genome sequence of race 2 strain 4287 (*Fusarium oxysporum* Sequencing Project; Broad Institute of Harvard and MIT (http://www.broad.mit.edu). Molecular weight markers are indicated on the left (in kb).

In an initial analysis of the xylem sap proteome of tomato plants infected with Fol race 1 using 2-D gel electrophoresis and mass spectrometry, three small secreted proteins of Fol were identified in addition to Avr3 (Six1), named Six2, Six3 and Six4, and their genes cloned [18]. We now find that one of these, Six4, is not secreted by Fol race 2 (Fig. 1). For reasons detailed below, we now call this protein Avr1. Like the *AVR3* (*SIX1*) gene, *AVR1* is surrounded by repetitive elements (Fig. 2A). In all of the race 1 strains we examined, PCR experiments detected the presence of *AVR1* and no sequence polymorphism was detected in the coding regions of seven isolates from different clonal lines (see [9] for the list of strains; 17 of these are race 1, 23 are race 2 or 3). *AVR1* was not detected in race 2 or 3 strains by PCR nor is *AVR1* present in the genome sequence of the race 2 strain 4287 (*Fusarium oxysporum* Sequencing Project; Broad Institute of Harvard and MIT (http://www.broad.mit.edu)). Absence of *AVR1* or closely related genes in the race 2 and race 3 strains used in this study was confirmed by DNA gel blot analysis (Fig. 2B, lanes 4 and 7, respectively).

**Figure 3:**
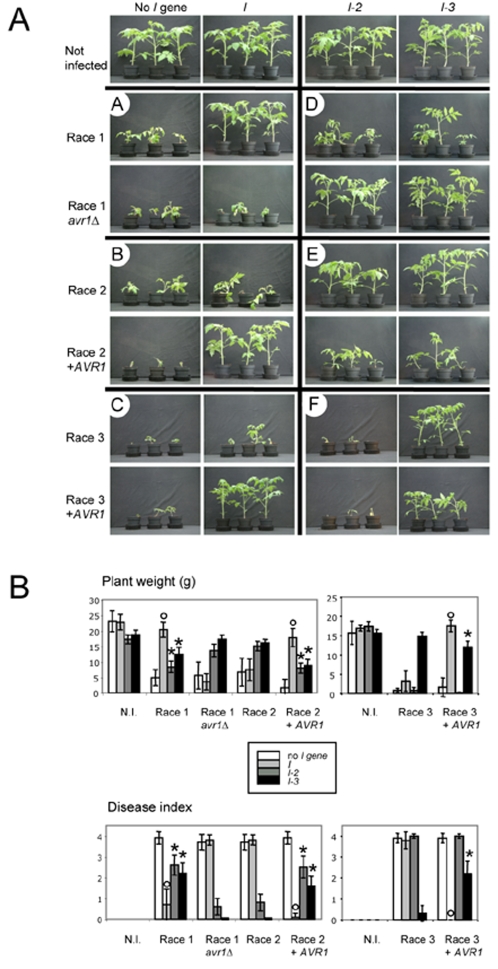
Avr1 suppresses *I-2* and *I-3* mediated resistance. Ten day old seedlings of tomato were inoculated with a fungal spore suspension and disease was scored after three weeks. Tomato lines carrying only a single resistance gene or no resistance gene were used to determine the effect of Avr1 on the activity of each resistance gene (see Materials and methods for description of plant lines). All lines were inoculated with the following Fol strains: race 1 (strain Fol004), race 2 (strain Fol007), race 3 (strain Fol029), race 1 *avr1*Δ (Fol004 with *AVR1* deleted by gene replacement), race 2+*AVR1* (Fol007 transformed with *AVR1*; similar virulence patterns were obtained with six independent transformants ) and race 3+*AVR1* (Fol029 transformed with *AVR1*; similar virulence patterns were obtained with four independent transformants). A) Representative plants are shown three weeks after infection. Panel A shows that loss of *AVR1* leads to breaking of *I*-mediated resistance. Panel B and C show that gain of *AVR1* triggers *I*-mediated resistance. Panel D shows that loss of *AVR1* leads to loss of virulence on *I-2* and *I-3*-containing plant lines. Panels E and F show that gain of *AVR1* by race 2 or race 3 leads to virulence on *I-2* and *I-3*-containing plant lines. B): Quantification of disease assays. The outcomes of the disease assays depicted in (A) were quantified in two ways: 1) average plant weight above the cotyledons and 2) phenotype scoring according to a disease index ranging from zero (no disease) to four (heavily diseased or dead). Error bars indicate the 95% confidence interval of the mean. Interactions where Avr1 induces *I*-mediated resistance are indicated with a circle. Interactions where Avr1 suppresses *I-2* or *I-3* are indicated with an asterisk. N.I: not infected.

To test whether *AVR1* is indeed responsible for avirulence of Fol on plants carrying the *I* gene, we created an *AVR1* gene knock-out in a race 1 strain (Fol004) through *Agrobacterium*-mediated transformation (Fig. 2). For the *AVR1* gene, the frequency of homologous recombination leading to gene knock-out turned out to be extremely low, with only a single knock-out mutant obtained out of ∼200 transformants (Fig. 2B, lane 2). A disease assay with this mutant (*avr1*Δ) confirmed that indeed deletion of *AVR1* leads to breaking of *I*-mediated disease resistance (Fig. 3A, panel A, quantified in Fig. 3B). Re-introduction of *AVR1* in the *avr1*Δ strain (Fig. 2B, lane 3) restored the original avirulence phenotype (results not shown). In addition, we found that disease resistance conferred by the unlinked *I-1* gene in tomato also depends on recognition of Avr1, since the *avr1*Δ strain (but not its parental strain) is virulent on a plant line carrying *I-1* (line 90E402F, results not shown). This suggests that *I* and *I-1* express the same resistance specificity.

To confirm that the *AVR1* gene is sufficient to trigger recognition by the *I* gene, we transformed *AVR1* to a race 2 strain (Fol007) and a race 3 strain (Fol029) that do not contain *AVR1* (Fig. 2B, lanes 4–9) and are virulent on *I*-containing tomato lines. Ten independent transformants (six of race 2 and four of race 3) containing *AVR1* were unable to cause disease on *I*-containing plants (Fig. 3A, panels B and C, quantified in Fig. 3B), confirming the avirulence character of *AVR1*. In contrast to Avr3 [9], Avr1 is dispensable for full virulence towards plants that do not contain *R* genes against Fol (results not shown).

### Avr1 suppresses *I-2* and *I-3*-mediated disease resistance

Although all Fol strains possess an intact *AVR3* gene, most race 1 strains nevertheless cause disease on plants carrying only the *I-3* gene [9]. One explanation for this is that Avr1 itself is involved in suppression of *I-3* mediated disease resistance. To test this, we inoculated a plant line containing only the *I-3* gene with the set of Fol strains described above. The results clearly show that Avr1 indeed has this suppressive activity: deletion of *AVR1* in race 1 leads to loss of virulence towards *I-3* plants (Fig. 3A, panel D, quantified in Fig. 3B), while introduction of *AVR1* in race 2 or race 3 leads to gain of virulence towards *I-3* plants (Fig. 3A, panels E and F, quantified in Fig. 3B). Furthermore, we discovered that Avr1 also suppresses *I-2*-mediated disease resistance (Fig. 3A, panels D and E, quantified in Fig. 3B). This means that the ability of some race 1 strains to cause disease on *I-2* plants, as observed earlier [10], is likely to be caused by suppression of *I-2* rather than loss of *AVR2*. In accordance with earlier observations using *I-3* plants [9], we found that virulence due to suppression of *I-2* and *I-3* is partial compared to strains lacking the corresponding *AVR* gene (Fig. S1). It should be noted that not all race 1 strains are virulent on *I-2* and/or *I-3* plants [9],[10], even though all contain *AVR1* with identical sequences (results not shown). Apparently, suppression of *R* gene-based immunity by Avr1 is dependent on unknown factors in the genetic background of the fungus. Since suppression works in Fol007 (race 2) and Fol029 (race 3), the genetic background in which *AVR1* is effective is not restricted to race 1 strains.

### Possible function of Avr1

**Figure 4:**
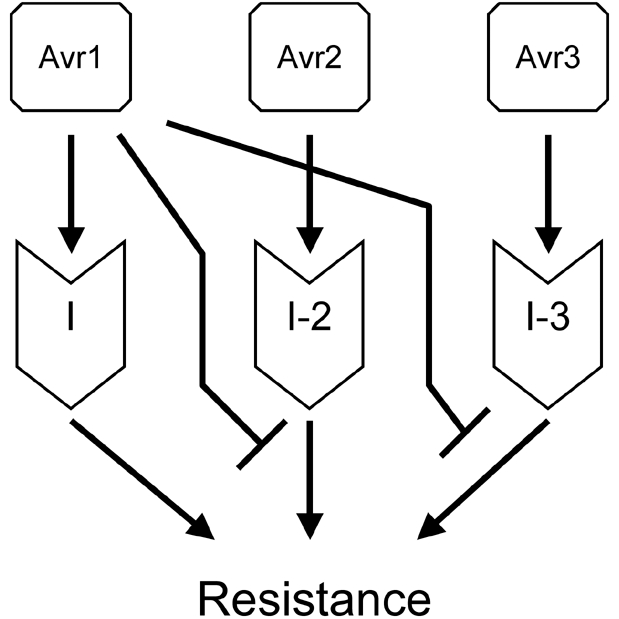
Schematic summary of the interactions between Fol Avr proteins and tomato resistance (I) proteins. Arrows signify activation, lines ending in a cross bar signify suppression. Avr1 is synonymous to Six4, Avr3 is synonymous to Six1.

Our observation that Avr1 is not required for virulence to plants without *I* genes may be due to the existence of other effectors that are redundant for such an activity. Alternatively, the role of Avr1 is restricted to the suppression of *I-2* and *I-3*-mediated disease resistance. A mechanistic explanation for the latter role could be that Avr1 interferes directly with Avr2 and Avr3. However, at least Avr3 accumulates in xylem sap and remains unaltered in the presence of Avr1 [9],[18]. A direct interaction between the two proteins could also not be demonstrated *in vitro* by pull down experiments (results not shown). Unlike bacteria, pathogenic fungi are not known to inject proteins directly into plant cells, but many are known to secrete small, frequently cysteine-rich, but otherwise unrelated proteins during colonization of plants [5]. Avr1, like Avr3, falls within this group, the predicted mature protein having 184 residues including 6 cysteines and lacking homology to other proteins [18]. The mode of action of most of these small secreted proteins has remained unclear. Molecular targets have been described for Avr2 and Avr4 from the leaf mold *Cladosporium fulvum*: Avr2 is a protease inhibitor [19] while Avr4 binds chitin in the fungal cell wall and protects it against attack by plant chitinases [20]. These two proteins act in the apoplast to enhance fungal virulence, but others act inside plant cells [4]. Uptake from the apoplast by plant cells has been shown directly for ToxA, a small secreted protein that acts as a host-selective toxin [21]. This may also occur with Avr2, since I-2 is a cytoplasmic protein [15]. Avr1, then, may interfere with the uptake of Avr2 and Avr3. Alternatively, it may be taken up itself and interfere with I-2 and I-3 or with signal transduction processes downstream of these R proteins (Fig. 4).

### Implications for the evolution of Avr-*R* gene interactions

Suppression of effector-triggered (*R* gene-mediated) immunity has been observed in bacteria [3],[22],[23]. In plant pathogenic fungi, suppression of avirulence by unlinked loci has been demonstrated by genetics in rust fungi [24]. In the flax rust fungus, two dominant alleles or tightly linked genes at the *I* (“inhibitor”) locus suppress – sometimes partially – either one (*M1*) or several (*M1*, *L1*,*7*,*8*,*10*) *R* genes out of 30 against flax rust [24],[25]. The flax rust inhibitor locus is not itself linked to avirulence. Here, we report the identification of a fungal avirulence factor that suppresses disease resistance conferred by two *R* genes.

Interpreting this phenomenon in terms of molecular arms races between plants and their pathogens [1], we envisage the following scenario. During evolution of the tomato-Fol pathosystem, *I-2* and *I-3* have evolved to recognize, respectively, Avr2 and Avr3. Since Avr3 is required for full virulence of Fol, evasion of *I-3* recognition through loss of the *AVR3* gene would entail a serious fitness penalty. This explains why all Fol strains analysed so far retained *AVR3* [9],[26]. Point mutations in *AVR3* preventing recognition have not been found either [9]. A possible explanation for this is that the I-3 protein operates in accordance with the guard model, in which not the Avr3 protein itself but the effect it has on its virulence target is recognized [27]. In any case, Fol has (partially) regained virulence towards *I-3*-containing plants by acquisition of *AVR1*, which, as shown here, suppresses the function of *I-3*. Subsequently, tomato responded to this ‘invention’ with the employment of the *I* gene, or the unlinked *I-1* gene, to specifically recognize and respond to Avr1. Apparently, *I* and *I-1* are themselves insensitive to the suppressive effect of Avr1 (Fig. 4).

The agricultural ‘arms race’ between Fol and tomato is different from the natural one because it is dictated by successive *R* gene deployment in commercial cultivars [8]. The *I* gene from the wild tomato relative *Solanum [Lycopersicon] pimpinellifolium* was the first *R* gene to be introgressed into tomato cultivars to resist Fusarium wilt in the 1940s [12]. At that time, Fol strains without Avr1 may already have been present in some locations, since *I*-breaking race 2 strains were quickly discovered [28] even though major outbreaks did not occur before 1960 [29]. The *I-2* gene, also from *S. pimpinellifolium* and directed against Avr2, was introduced in commercial cultivars in the 1960s to protect tomato against Fol race 2 [29],[30]. The combination of *I* and *I-2* was effective for about two decades until the appearance of race 3 in both Australia and North America [31], which probably emerged from a race 2 background through selection for loss or mutation of *AVR2*. To combat race 3, the *I-3* gene was introgressed from *S. pennellii* [31]. From the results presented here, we deduce that the combination of *I* (or *I-1*) and *I-3* may yield durable resistance of tomato to Fusarium wilt disease of tomato, since *I-3* is directed against a virulence factor (Avr3) and *I* (and *I-1*) against the suppressor of *I-3* (Avr1).

The molecular toolbox that is now gradually filling up (Avr1, Avr3, I-2) will help us to define host targets and evolutionary bottlenecks that govern the arms race in the Fol-tomato pathosystem. It also may allow development of new strategies for breeding plants with durable resistance against fungal pathogens.

## Materials and Methods

### Plant lines and fungal strains

The following tomato lines were used (Fol resistance genes between brackets): GCR161 (*I*) [32], 90E402F (*I-1*) [31],[33]; 90E341F (*I-2*) [29] and E779 (*I-3*) [31], C32 (no *I* gene) [32]. The following Fol strains were used: Fol004 (race 1), Fol002 (race 2), Fol007 (race 2), Fol029 (race 3), Fol004avr1Δ (Fol004 with *AVR1* deleted by gene replacement), Fol004avr1Δ+*AVR1* (Fol004avr1Δ transformed with *AVR1*), Fol007+*AVR1* (Fol007 transformed with *AVR1*), Fol029+*AVR1* (Fol029 transformed with *AVR1*). See Rep *et al.* (2005) [9] for a more detailed description of the wild type Fol strains.

### Xylem sap proteome analysis

Proteins present in xylem sap of tomato plants infected with Fol were isolated and separated with 2-dimensional gel electrophoresis as described earlier [18], using for the first dimension an Immobiline DryStrip of 13 cm, pH 6–11 NL (Amersham Biosciences).

### Disease assays

Ten day old seedlings of tomato were inoculated with a fungal spore suspension and disease was scored after three weeks as described earlier [17]. The outcome of the disease assays was quantified in two ways: 1) average plant weight above the cotyledons and 2) phenotype scoring according to a disease index ranging from zero (no disease) to four (heavily diseased or dead) [17].

### *AVR1* disruption and complementation constructs

The *AVR1* disruption construct was made by PCR amplification of *AVR1* upstream and downstream sequences for homologous recombination, and their insertion in front of and behind the hygromycin resistance gene in the vector pRW2h (see below): an upstream fragment, from 714 bp to 1 bp upstream of the start codon, was cloned into pRW2h between the *Pac*I and *Kpn*I sites, and a downstream fragment, from 375 bp after the start codon to 537 bp downstream of the stop codon, was cloned into pRW2h between the *Xba*I and *Bss*HII sites (see Fig. 2A for location of the primers). The construct for complementation was made by amplification of a *AVR1* expression cassette from 714 bp upstream of the start codon to 537 bp downstream of the stop codon (Fig. 2A), which was inserted between the *Xba*I and *Stu*I sites of pRW1p (see below). Transformation of these constructs to Fol was done with *Agrobacterium* as described earlier [34].

pRW2h is a binary vector for *Agrobacterium*-mediated transformation of fungi. It was made through insertion of a *Nhe*I-*Xba*I fragment from pAN7.1, carrying the hygromycin resistance gene *hph* under control of the *Aspergillus* (*Emericella*) *nidulans gpd* promoter and *trpC* terminator [35], into the unique *Xba*I site of pPZP-201BK [36]. Similarly, pRW1p was derived from pPZP-201BK through insertion of a *Nhe*I-*Xba*I fragment from pAN8.1 [35] carrying the phleomycin resistance gene *ble* under control of the same *gpd* promoter and *trpC* terminator.

### Southern blotting

Genomic DNA of *F. oxysporum* was isolated according to Raeder and Broda [37], digested with *Hin*dIII and *Bam*HI, separated in a 1% agarose gel and blotted to Hybond N+ according to Sambrook *et al.* [38]. The probe containing the *AVR1* ORF and 3′ sequences (1402 bp, Fig. 2A) was generated by PCR and contains sequences from 72 bp upstream to 537 bp downstream of the ORF. The probe was radioactively labelled with α^32^P dATP using the DecaLabel™ DNA labeling kit from MBI Fermentas (Vilnius, Lithuania). Hybridization was done overnight at 65°C in 0.5M phosphate buffer pH 7.2 containing 7% SDS and 1 mM EDTA. Blots were washed at 65°C with 0.2 X SSC, 0.1% SDS. The position of sequences hybridizing to the probe were visualized by phosphoimaging (Molecular Dynamics).

### Accession numbers

The *AVR1* (*SIX4*) locus: AM234064

The Avr1 (Six4) protein: CAJ84000

## Supporting Information

Figure S1 Suppression of *I-2* and *I-3* is partial. Ten day old seedlings of tomato were inoculated with a fungal spore suspension and disease was scored after three weeks as described earlier. Tomato lines carrying only *I-2* (90E341F) or *I-3* (E779) were either mock-inoculated (A,B) or inoculated with race 1 strain Fol004 that suppress *I-2* and *I-3* (C, D) or with strains that avoid recognition by *I-2* or *I-3* through absence of the corresponding *AVR* gene (E, F). In (E), race 3 strain Fol029 (no *AVR2*) was used. In (F), Fol004 avr3Δ (race 1 strain Fol004 with *AVR3* (*SIX1*) deleted by gene replacement) was used. Representative plants are shown three weeks after infection. Note that although *AVR3* is required for full virulence towards susceptible plants of three weeks and older, *AVR3* is not required for virulence in the seedling assay used here, allowing assessment of the effectiveness of individual *R* genes [9].
(5.94 MB TIF)
