# Supplementary figures and images for: Suppression of Plant Resistance Gene-Based Immunity by a Fungal Effector
Source: PLoS Pathog. 2008 May 9;4(5):e1000061. doi: 10.1371/journal.ppat.1000061 (PMC2330162; doi:10.1371/journal.ppat.1000061)

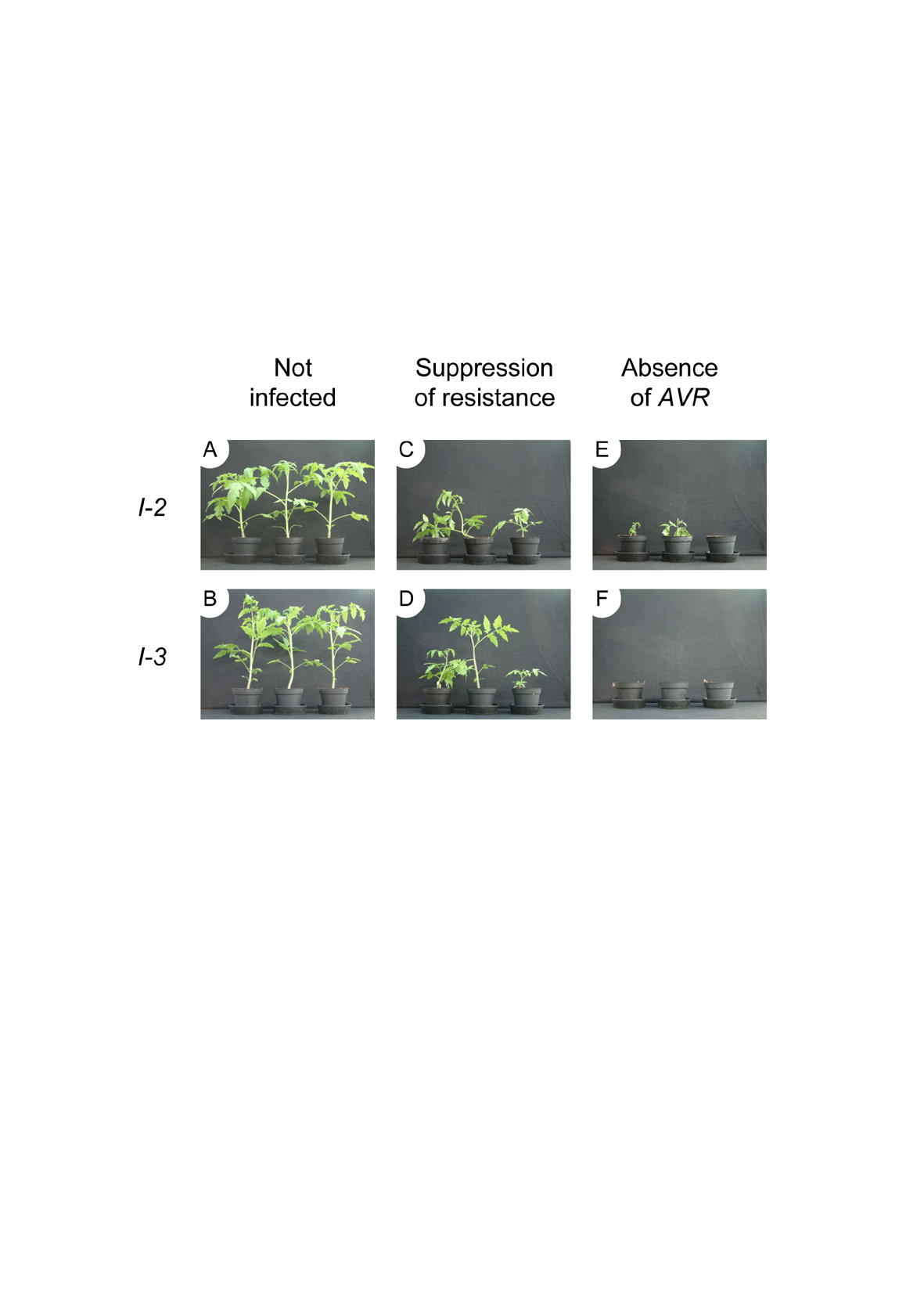

Supplement: Figure S1 — Suppression of I-2 and I-3 is partial. Ten day old seedlings of tomato were inoculated with a fungal spore suspension and disease was scored after three weeks as described earlier. Tomato lines carrying only I-2 (90E341F) or I-3 (E779) were either mock-inoculated (A,B) or inoculated with race 1 strain Fol004 that suppress I-2 and I-3 (C, D) or with strains that avoid recognition by I-2 or I-3 through absence of the corresponding AVR gene (E, F). In (E), race 3 strain Fol029 (no AVR2) was used. In (F), Fol004 avr3Δ (race 1 strain Fol004 with AVR3 (SIX1) deleted by gene replacement) was used. Representative plants are shown three weeks after infection. Note that although AVR3 is required for full virulence towards susceptible plants of three weeks and older, AVR3 is not required for virulence in the seedling assay used here, allowing assessment of the effectiveness of individual R genes [9]. (5.94 MB TIF) [file ppat.1000061.s001.tif]
